# Crystal Structures Reveal the Multi-Ligand Binding Mechanism of *Staphylococcus aureus* ClfB

**DOI:** 10.1371/journal.ppat.1002751

**Published:** 2012-06-14

**Authors:** Hua Xiang, Yue Feng, Jiawei Wang, Bao Liu, Yeguang Chen, Lei Liu, Xuming Deng, Maojun Yang

**Affiliations:** 1 Key Laboratory for Protein Sciences of Ministry of Education, Tsinghua-Peking Center for Life Sciences, School of Life Sciences, Tsinghua University, Beijing, China; 2 Department of Veterinary Pharmacology, College of Animal Science and Veterinary Medicine, Jilin University, Changchun, China; 3 Department of Vascular Surgery, Peking Union Medical College Hospital, Beijing, China; 4 Department of Chemistry, Tsinghua University, Beijing, China; University of Michigan, United States of America

## Abstract

*Staphylococcus aureus* (*S. aureus*) pathogenesis is a complex process involving a diverse array of extracellular and cell wall components. ClfB, an MSCRAMM (Microbial Surface Components Recognizing Adhesive Matrix Molecules) family surface protein, described as a fibrinogen-binding clumping factor, is a key determinant of *S. aureus* nasal colonization, but the molecular basis for ClfB-ligand recognition remains unknown. In this study, we solved the crystal structures of apo-ClfB and its complexes with fibrinogen α (Fg α) and cytokeratin 10 (CK10) peptides. Structural comparison revealed a conserved glycine-serine-rich (GSR) ClfB binding motif (GSSGXGXXG) within the ligands, which was also found in other human proteins such as Engrailed protein, TCF20 and Dermokine proteins. Interaction between Dermokine and ClfB was confirmed by subsequent binding assays. The crystal structure of ClfB complexed with a 15-residue peptide derived from Dermokine revealed the same peptide binding mode of ClfB as identified in the crystal structures of ClfB-Fg α and ClfB-CK10. The results presented here highlight the multi-ligand binding property of ClfB, which is very distinct from other characterized MSCRAMMs to-date. The adherence of multiple peptides carrying the GSR motif into the same pocket in ClfB is reminiscent of MHC molecules. Our results provide a template for the identification of other molecules targeted by *S. aureus* during its colonization and infection. We propose that other MSCRAMMs like ClfA and SdrG also possess multi-ligand binding properties.

## Author Summary

*Staphylococcus aureus* (*S. aureus*), an important opportunistic pathogen, is a major threat to humans and animals, causing high morbidity and mortality worldwide. It is responsible for a variety of infections ranging from mild superficial infections to severe infections such as infective endocarditis, septic arthritis, osteomyelitis and sepsis. Such infections are of growing concern due to the increasing antibiotic resistance of *S. aureu*s. In order to understand the mechanism of the *S. aureu*s pathogenesis, we studied one of the bacterial surface proteins clumping factor B (ClfB) bound by the fibrinogen α (Fg α) and cytokeratin 10 (CK10). From analyses of the high resolution crystal structures we found that the ClfB-binding peptides harbor a stretch with consensus sequence (GSSGXGXXG) that is also conserved in Engrailed protein, TCF20 and Dermokines. The interaction between ClfB and a dermokine-derived peptide was demonstrated using binding assays. Consistent with a role of ClfB in the inflammatory responses induced by *S. aureus*, expression of dermokines is predominant in epithelial tissues and upregulated in inflammatory diseases. The data presented in this study raise a possibility that multiple human proteins are targeted by ClfB during *S. aureus* infection. The multi-ligand binding feature of ClfB would be valuable for developing new therapeutic strategies.

## Introduction

*Staphylococcus aureus* (*S. aureus*), an important opportunistic pathogen, is a major threat to humans and animals causing high morbidity and mortality worldwide. It is responsible for a variety of infections ranging from mild superficial infections to severe infections such as infective endocarditis, septic arthritis, osteomyelitis and sepsis [1]. Such infections are of growing concern because of the increasing antibiotic resistance of *S. aureu*s [2], [3]. Multiple sites within the body can be colonized, including the perineum and the axilla, but the most frequent site of the carriage is the moist squamous epithelium of the anterior nares. Moreover, the organism can be disseminated from a superficial site via the bloodstream to internal organs where it can set up a metastatic focus of infection. Approximately 80% of invasive *S. aureus* infections are autologous in that they are caused by strains carried in the patient's nose prior to illness [4], [5].

The ability of *S. aureus* to cause diseases has been generally attributed to two classes of virulence determinants: cell wall-associated proteins and extracellular protein toxins. The initial step in pathogenesis is often cell adhesion, mediated by surface adhesins called MSCRAMMs (Microbial Surface Components Recognizing Adhesive Matrix Molecules) [6], [7]. To date, *S. aureus* is known to express more than 20 different potential MSCRAMMs [8], [9].

SD-repeat-containing (Sdr) proteins are members of the MSCRAMM family, including clumping factor A (ClfA), ClfB, SdrC, SdrD and SdrE of *S. aureus* and SdrF and SdrG of *S. epidermidis*. The Sdr proteins are characterized by the presence of an R region composed largely of repeated SD dipeptides [10]. They exhibit a comparable structural organization including an N-terminal secretory signal sequence followed by a ligand-binding A region and a dipeptide repeat region (R) composed mainly of aspartate and serine residues. The LPXTG cell wall-anchoring motif (W) immediately follows the SD-repeat region and is followed by a hydrophobic membrane-spanning domain (M) and a short positively charged cytoplasmic tail (C). Despite their conserved structural organization, the Sdr proteins are not closely related in sequence, with only 20 to 30% identical amino acid residues in the ligand-binding A domain. This suggests that different Sdr proteins might play different roles in *S. aureus* pathogenesis [11].

ClfB is one of the best characterized surface proteins on *S. aureus* during the past decade [12]–[18]. The multi-functional characteristics are quite unique to this adhesin, unlike ClfA and SdrG that have been shown to bind only to fibrinogen [19]–[21]. ClfB plays a key role in establishing human nasal colonization by binding to the human type I cytokeratin 10 (CK10) expressed on squamous epithelial cells [17], [18], [22], [23]. Consistently, recent studies have shown that the immunization of mice with ClfB reduces nasal colonization [24]. As a bifunctional MSCRAMM, ClfB also binds to fibrinogen α (Fg α), which is assumed to be significant in platelet activation and aggregation and has been shown to contribute to the pathogenesis of experimental endocarditis in rats [7], [17], [25], [26]. Unlike ClfA, FnBPA and FnBPB, which bind to the γ chain of fibrinogen, ClfB binds to repeat 5 (NSGSSGTGSTGNQ) of the flexible region of its α chain [15], [16], [27]. The repeat may form a loop, similar to the Tyr-(Gly/Ser)*_n_* Ω loops present in the C-terminus of CK10, to which ClfB also binds [18]. Fg α and CK10 harbor the same or overlapping binding sites on ClfB [18], but the detailed mechanism of ClfB recognition of Fg α and CK10 is unclear.

Structural studies suggest that ClfA and SdrG have different ligand binding characteristics and mechanisms [21], [28], although the structural organizations of the adhesion domains of these two MSCRAMMs are very similar. A “dock, lock, and latch” (DLL) model was proposed for SdrG-ligand recognition, where SdrG adopts an open conformation that allows the Fg ligand to access a binding trench between the N2 and N3 domains [21]. In ClfA, however, the cavity is preformed in a stabilized closed configuration, into which the C-terminal of the γ chain of fibrinogen threads. Therefore, the ClfA-Fg binding mechanism was proposed to be “Latch and Dock” [28].

Here we solved the crystal structures of the apo-ClfB adhesive domain and its complexes with peptides derived from Fg α and CK10. Our structures showed that ClfB recognizes its ligands in a similar manner with the DLL model. A previous study on the structures of ClfB complexed with Fg α and CK10 peptides suggested that the conserved peptide-derived motif (GSSGXG) is required for their binding to ClfB [29]. The data presented in the present study, however, support a minimal nine amino acids Gly-Ser-Rich (GSR) motif that is necessary and sufficient for binding to ClfB. Human genome mining using the motif as a template identified several candidates including Engrailed protein, TCF20 and Dermokine as potential ClfB-binding proteins. Interaction of Dermokine with ClfB was confirmed by biochemical and structural studies, which demonstrate that nearly identical mechanisms are utilized by ClfB to recognize its binding partners. Our data not only provides insights into the ligand binding mechanism of ClfB but also raises the possibility that ClfB targets multiple substrates during *S. aureus* infections. These results would be valuable for the development of new therapeutic strategies.

## Results

### Structure of apo-ClfB_(197–542)_

**Figure 1:**
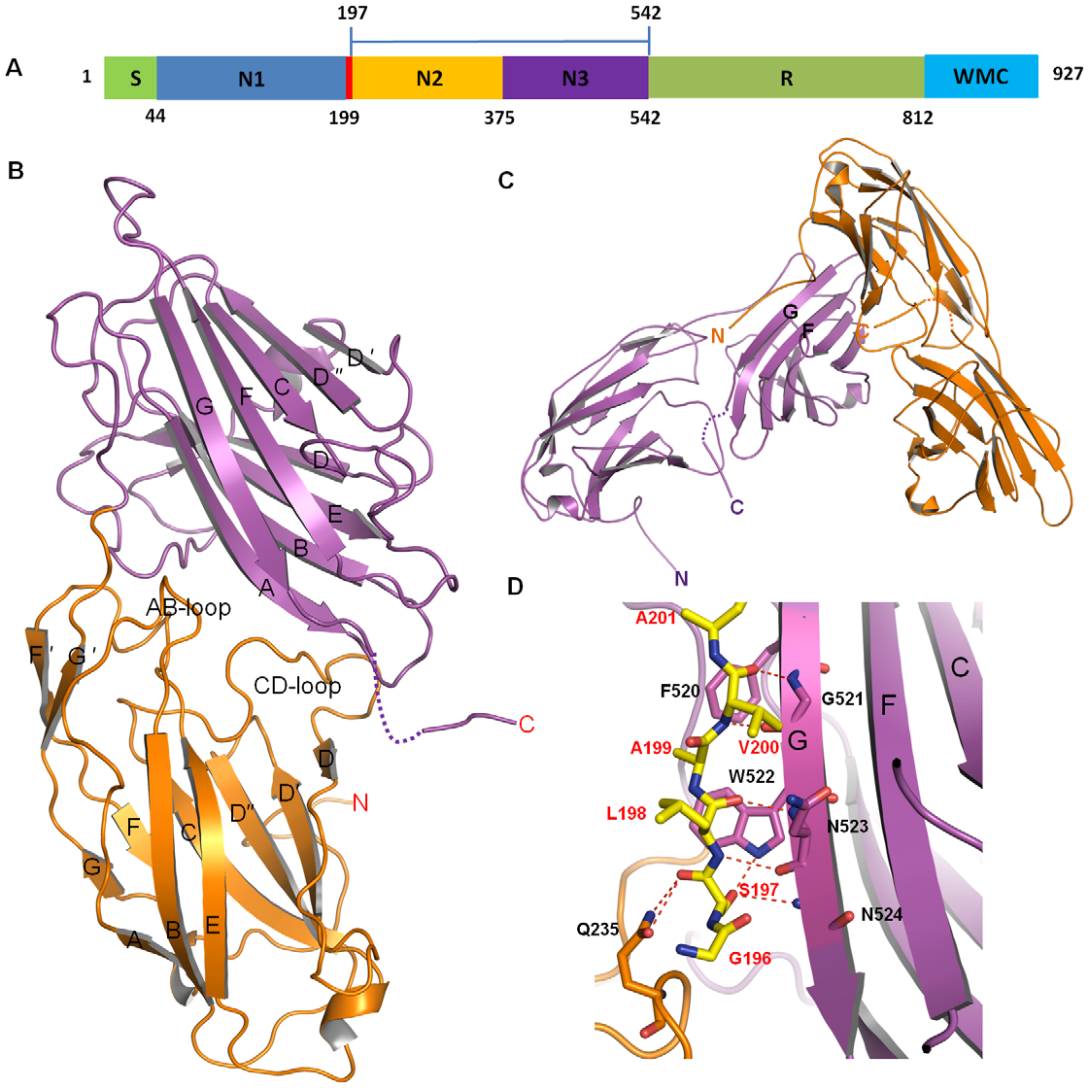
Crystal structure of apo-ClfB_(197–542)_. A. Domain organization of ClfB. The numbers of the amino acid residues identifying the boundaries between adjacent domains are indicated below. S, signal sequence; N1-3, N-terminal fibrinogen binding region; R, serine-aspartate repeat region; W, wall-spanning domain; M, membrane anchor; C, cytoplasmic positively charged tail. The N2 and N3 domains were used in crystallization of the ClfB_(197–542)_-peptide complexes. B. Ribbon representation of the structure of apo-ClfB_(197–542)_, with its N and C terminus indicated. The N2 and N3 domains are shown in orange and magenta, respectively. The strands and loops are marked. C. Ribbon representation of the two symmetry-related molecules in the unit cell, shown in orange and magenta, respectively. The N and C termini of both molecules are indicated. D. Closer view of the interaction between the two symmetry-related molecules. The N-terminus of one molecule (amino acids 196–201) is shown as sticks and the other one is colored in magenta as in (B). The amino acids from both molecules are marked in red and black characters, respectively. The hydrogen bonds are shown as red dashed lines.

Previous studies indicated that a segment of ClfB containing N2 and N3 regions (Figure 1A) is sufficient for recognition of Fg α and CK10 [18], [27], [29]. We therefore cloned the segment encoding the two regions (amino acids 197 to 542) of the ClfB protein from *S. aureus* and purified the protein from *E. coli* for our structural studies. The structure of the ClfB_(208–540)_-Fg α_(316–328)_ complex was solved by a Se-Met derived protein and was used as a starting model for determination of the other structures by the molecular replacement method (Table 1).

**Table 1:** Statistics of data collection and structure refinement.

Parameter	Fg α bound	CK10 bound	Dermokine bound	Peptide free
Data collection	Se-SAD	Native	Native	Native
Space Group	P3_1_2_1_	P3_1_2_1_	P3_1_2_1_	P 4_3_ 2_1_ 2
Unit Cell (Å)	70.98, 70.98, 174.91	70.33, 70.33, 177.15	70.08, 70.08, 175.76	94.42, 94.42,86.71
Wavelength (Å)	0.979	0.979	0.919	0.979
Resolution (Å)	1.92 (1.99-1.92)	2.3 (2.34-2.3)	2.5 (2.59-2.5)	2.51 (2.6-2.51)
Rsym* (%)	9.9(68)	8.6(50)	7.8(54)	7.5(53.1)
I/sigma	56.5(1.75)	28(2.5)	22.9(2.1)	45.6(10.1)
Completeness (%)	99.5(98.1)	99.6(99.5)	98.3(88.1)	100(100)
Redundancy	8.2(7.6)	10.2(7.9)	8.5(5.9)	14.4(9.9)
Wilson B factor (Å^2^)	40.6	38.7	61.3	52.1
**SAD phasing**				
Anomalous scatterers	3 Se			
Figure-of-merit (FOM)	0.387			
FOM after DM	0.687			
FOM after phase combination	0.788			
**Refinement**				
R factor#	0.1792	0.2204	21.95	23.29
R free	0.2145	0.2546	25.43	28.87
No. atoms	2899 protein atoms+117 H_2_O+2 Mg^2+^	2593 protein atoms+ 5 H_2_O+1 Mg^2+^	2640 protein atoms+11 H_2_O+2 Mg^2+^	2558 protein atoms+5 Mg^2+^
**B factors:**				
Overall	38.53	39.47	63.91	55.95
Main chain	36.45	37.11	62.64	53.21
Side chain	40.6	41.84	65.16	58.48
RMSD bond lengths	0.006	0.008	0.007	0.01
RMSD bond angles	0.998	1.168	1.168	1.169
**Ramachandran plot statistics (%)**				
Preferred regions	95.4	93.4	92.3	94.9
Allowed regions	4.4	5.2	6.8	3.9
Outliers	0.3	1.3	0.8	1.2
PDB code	4F27	4F1Z	4F20	4F24

**+:** Values in parentheses are for the highest resolution shell.

*: *R*_sym_ = Σ_h_Σ_i_|*I_h,i_*−*I_h_*|/Σ_h_Σ_i_ *I_h,i_*, where *I_h_* is the mean intensity of the *i* observations of symmetry related reflections of *h*.

# *R* = Σ|*F_obs_*−*F_calc_*|/Σ*F_obs_*, where *F_calc_* is the calculated protein structure factor from the atomic model (R_free_ was calculated with 5% of the reflections).

The apo-ClfB_(197–534)_ structure was solved at 2.5 Å resolution, consisting of residues Ser197-Ala534 (Figure 1B). No electron density was observed for the C-terminal eight residues in the apo-ClfB structure. The polypeptide chain of apo-ClfB_(197–534)_ is composed of two distinct domains N2 and N3, as previously described for other MSCRAMMs in *S. aureus* (Figure 1A) [30] . The N-terminal N2-domain contains 146 residues (amino acids 213–358) and the N3 domain 170 residues (amino acids 359–528). In the crystal structure, both N2 and N3 have two layers of β-sheets that pack tightly against each other (Figure 1B). In contrast, packing between the two domains is much looser, resulting in the formation of a large groove between them where presumably ligands bind. In N3 domain, strands A, B, E, and D form one of the two principal sheets, while strands D′, D″, C, F, and G on the opposite face present the other. Similar to the structures of other Fg-binding MSCRAMMs [21], [28], [29], [31], the structures of N2 and N3 display a typical Dev-IgG fold featured by the existence of the additional strands D′ and D″ as compared to the C-type IgG fold [30]. The structures of the N2 and N3 domains can be well superposed with an rms deviation of 0.98 Å for all Cα atoms. One structural difference between them, however, is the three-stranded β-sheet (A, B and E) on one side of N2 in comparison with a four-stranded β-sheet (D, F, C and G″) on its corresponding side in N3, as described in the structures of ClfA, SdrG and ClfB [21], [28], [29].

ClfB^Ser197^ and ClfB^Leu198^ or even a short N-terminally extended segment such as the unrelated His-tag were shown to be necessary to maintain the Fg binding activity of ClfB [16], though the mechanism of how the N-terminal segment of N2 participates in substrate binding is unclear. In the crystal structure of apo-ClfB, the N-terminus (Ser197-Ala201) of one ClfB_(197–542)_ molecule binds to the N3 domain of a symmetry-related ClfB molecule, forming a β-sheet together with the strand G (Figure 1C and Figure S1) mediated by 2 pairs of main-chain hydrogen bonds. Additional hydrogen bonds involving ClfB^Gln235^ and ClfB^Val200^ further contribute to the N-terminus-mediated interaction between ClfBs (Figure 1D). These interactions may act together to stabilize the G-strand of the N3 domain, thus maintaining its Dev-IgG fold and mimicking the transition state of ligand binding.

(All structural figures in this paper were generated with PyMOL [32]).

### Structure of the ClfB with Fibrinogen α (Fg α)_(316–328)_ and Type I Cytokeratin 10 (CK10)_(499–512)_ peptide complexes

**Figure 2:**
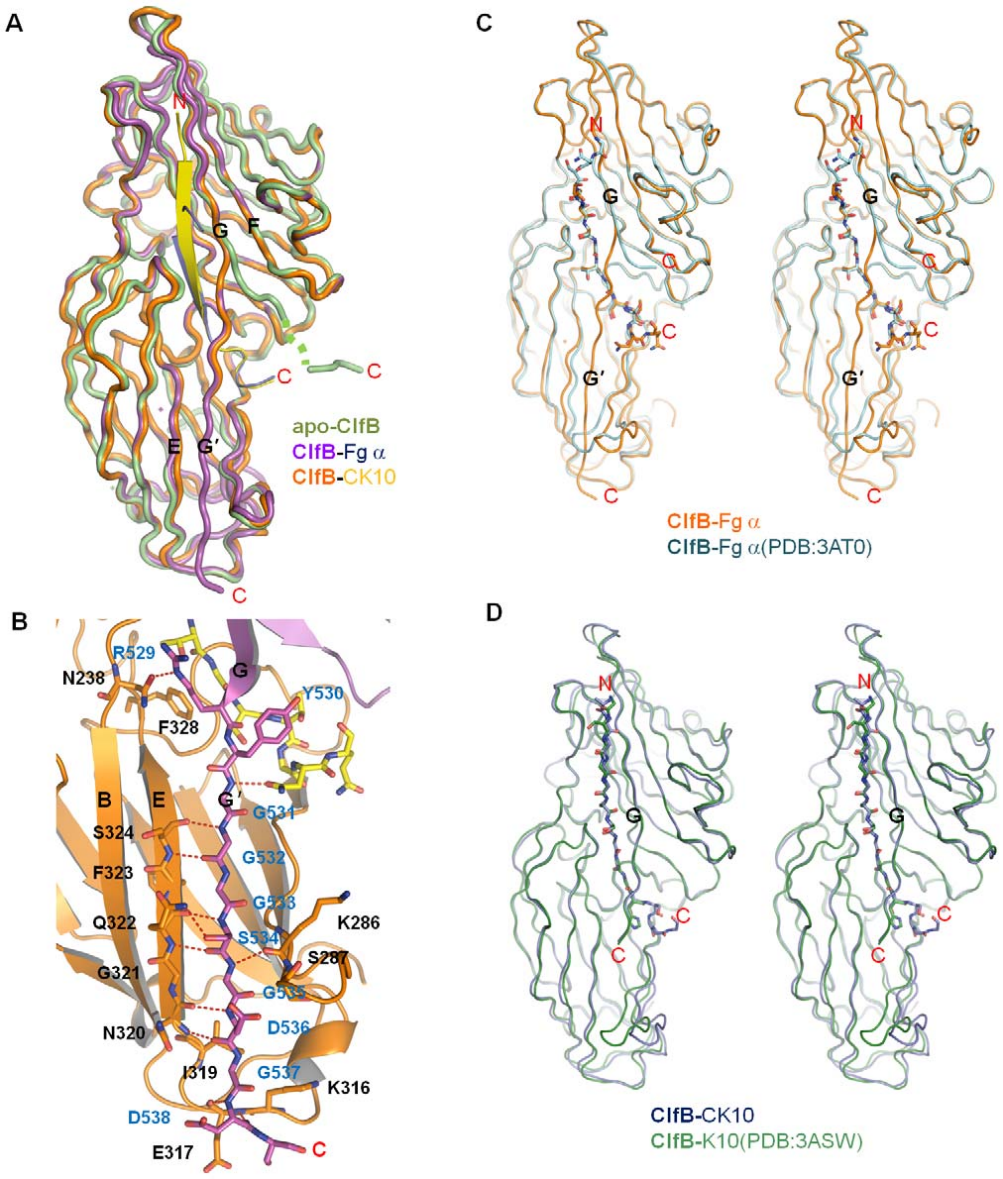
The ClfB-ligand binding is consistent with the DLL model. A. Ribbon representation of the superimposition of apo-ClfB, ClfB-Fg α and ClfB-CK10 complexes. The apo-ClfB is shown in limon. In the ClfB-Fg α complex, the protein and Fg α are shown in magenta and marine, respectively. In the ClfB-CK10 complex, ClfB and CK10 are shown in orange and yellow, respectively. B. Closer view of the interactions between the C-terminal G′ strand of N3 domain and N2 domain in the ClfB-Fg α complex. Residues involved in the interactions from both G′ strand and N2 domain are shown as sticks and marked by blue and black characters, respectively. The Fg α peptide is shown as sticks in yellow. C. Structural alignment of the ClfB-Fg α complex solved in this study and the one by V.Ganesh et al. (PDB entry: 3AT0) shown in stereo view, with RMSD 0.635 Å. The ClfB-Fg α complex in this study is shown in orange and the peptide is shown as sticks in the same color. The ClfB in the corresponding structure by V.Ganesh et al. is shown in cyan and Fg α is shown as sticks with its N and C termini marked. The C termini of ClfB in both structures and the G′ strand of ClfB in the current study are indicated. D. Structural alignment of the ClfB-CK10 complex solved in this study and the one by V.Ganesh et al. (PDB entry: 3ASW), with RMSD 0.479 Å. The ClfB-CK10 complex in this study is shown in marine and the peptide is shown as sticks with its N and C termini marked. The ClfB in the corresponding structure by V.Ganesh et al. is shown in lime and CK10 is shown as sticks. The C-termini of ClfB in both structures are indicated.

ClfB is a key adhesin mediating *S. aureus* adherence by binding to CK10 and Fg [18], [27]. To study the molecular mechanisms underlying ClfB-ligand recognition, we solved the crystal structures of ClfB_(208–542)_ in complex with CK10 (amino acids 499–512, referred as CK10_(499–512)_) or Fg α (amino acids 316–328, referred as Fg α_(316–328)_) at 2.3 Å and 1.92 Å, respectively (Figure 2A). The electron density unambiguously defines the existence of the peptides in the structures (Figure S2). In both complexes, the peptides adopt an extended conformation and are inserted into the tunnel formed between N2 and N3. Structure comparison revealed that the peptide binding induces an extension of β-strand G at its C-terminal side, which covers the bound peptides (Figure 2A and Figure S2). Similar structural features have also been observed in the structures of ClfA and SdrG complexed with their respective ligands (Figure S3) [21], [28]. Tight contacts between the peptide and the two domains in each complex result in extensive interactions, with a buried surface area of 966.6 Å^2^ in ClfB-Fg α_(316–328)_ and 1002.6 Å^2^ in ClfB-CK10_(499–512)_.

Structural comparison of the apo-ClfB and the two complexes shows that the RMSDs of the Cα atoms in ClfB are 0.46 Å and 0.49 Å respectively, indicating that the overall ClfB remains unchanged upon binding of the ligands (Figure 2A). Marked conformational changes, however, occur to the C-terminus of ClfB_(499–512)_ in both complexes. In ClfB-Fg α_(316–328)_, the residues ClfB^Arg529- Ser542^ that are disordered in the structure of apo-ClfB become well defined following Fg α_(316–328)_ binding. The distal C-terminus of ClfB_(197–542)_ forms a short β-strand G′, which forms a parallel β-sheet with the β-strand E from the N2 domain. The formation of the β-sheet is mediated by several main chain and side chain hydrogen bonds (Figure 2B). The ligand-induced stabilization of the C-terminal peptide of ClfB allows it to run across Fg α_(316–328)_ on the top. This binding mode is consistent with the DLL model as demonstrated in SdrG-Fg β complex [21], [28]. In contrast with Fg α_(316–328)_, the peptide CK10_(499–512)_ did not induce formation of the β-strand G′ in ClfB (Figure 2A). Nonetheless, the C-terminal portion of strand G that interacts with Fg α_(316–328)_ also becomes well defined and caps on the CK10_(499–512)_ peptide.

While we were preparing this manuscript, the structures of apo- and ligand binding ClfB were reported by V.Ganesh et. al [29]. Interestingly, the structural features we observed here are noticeably distinct from those of Fg α/CK10-ClfB complexes solved by them [29]. In both of their structures, particularly in the Fg α-ClfB complex, although the peptide adopts a conserved conformation as that in our structure, the C-terminus of the G-strand exhibits a different orientation and is not inserted into the N2 domain to form an extra strand G′ with the strand E, and thus the peptide is not locked in the groove between N2 and N3 (Figure 2C). In this way, their structures do not support the DLL model proposed based on the SdrG protein structure [21]. In addition, on peptide binding no rearrangement occurs to the loop between D and D′ in N2 (Figure 2C). Although the C-terminus of ClfB in the CK10-ClfB complexes has similar conformation as that in our structure, the D D′ loop in N2 domain shows no rearrangement, either (Figure 2D). The differences in the peptide conformations observed between our and Ganesh et al. works, could be attributed to the methodologies adopted in crystallization. While we co-purified the ClfB with the peptides to form a complex prior to crystallization, Ganesh et al. reported that they soaked the peptides into the apo-ClfB crystals [29]. In their structures, the conformational changes observed in our study to accommodate the peptide and then to lock it in place could have been hindered by crystal packing within the crystals.

In all, our structures strongly support the DLL model for ClfB-ligand binding. Briefly, “Dock” of the peptide triggers the rearrangement of the C-terminus of the N3 domain, allowing ClfB^Arg529^ to form a hydrogen bond with the ClfB^Asn238^ from N2 domain. This would result in “Lock” of the peptide into the substrate binding groove, whereas the strong interaction between G′ and the E strand of N2 can “Latch” the peptide (Figure 2B).

### Structural comparison of ClfB with ClfA and SdrG

**Figure 3:**
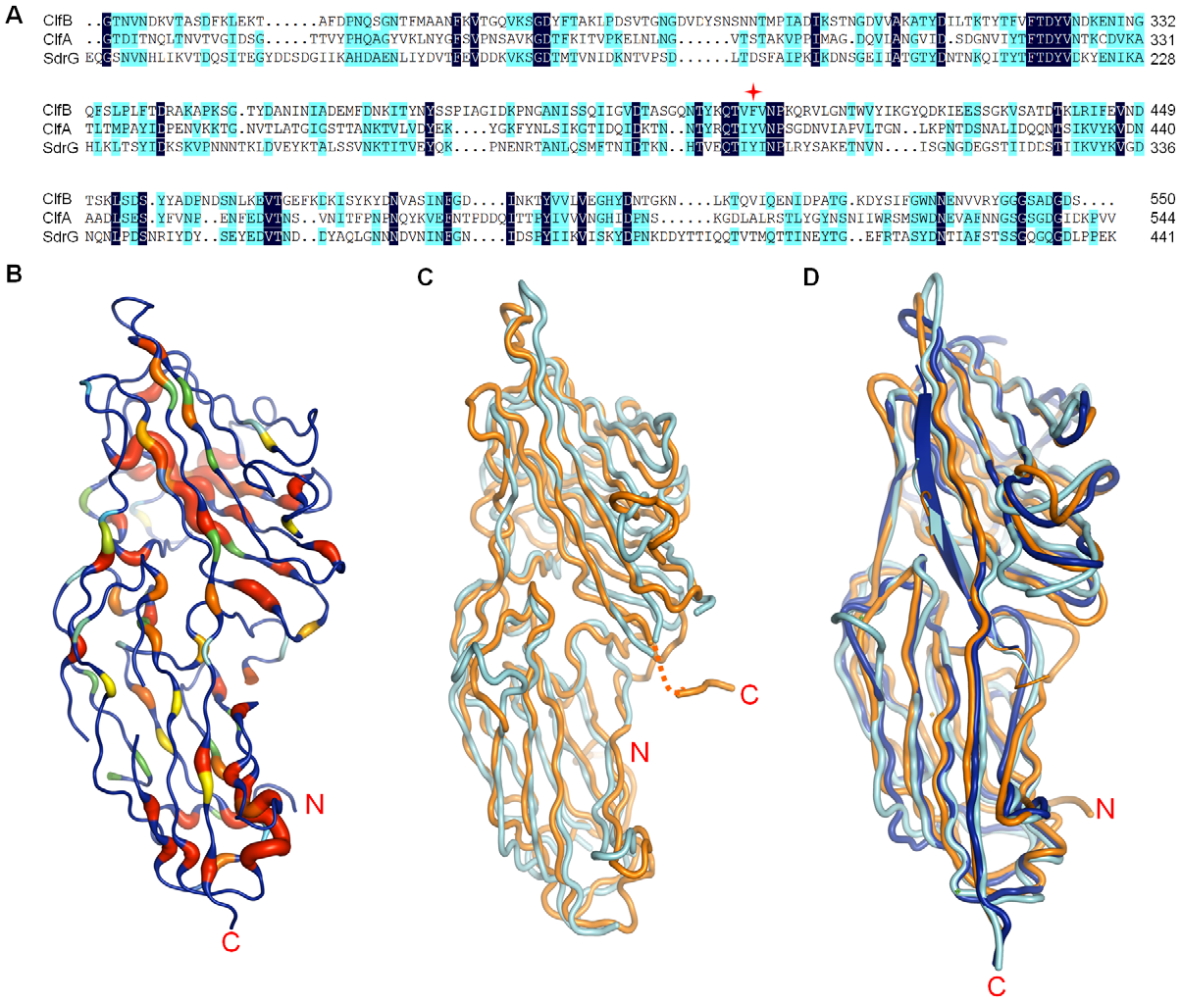
Structural aligment of ClfB, ClfA and SdrG. A. Sequence aligment of ClfB (amino acids 212–550), ClfA (amino acids 229–544) and SdrG (amino acids 117–441). Residues displaying 100% and 50% identity are shown in dark blue and light blue, respectively. F406 in ClfB is marked by red star. B. Ribbon representation of ClfB, with conserved residues colored from red to green following the order from highly conserved to less conserved. C. Superimposition of apo-ClfB and apo-SdrG, colored in orange and cyan, respectively. D. Superimposition of ClfB-Fg α, SdrG-Fg β and ClfA-Fg γ complexes. The SdrG-Fg β and ClfB-Fg α are colored as in Figure 3C. The ClfA-Fg γ complex is colored in blue.

In spite of the low identities in the amino acid sequences, the structures of ClfB, ClfA and SdrG exhibit high similarities (Figure 3). The most conserved residues are mainly located in the loop region of them (Figure 3B). Although the adherence domain organizations of ClfB, ClfA and SdrG and their ligand binding sites are conserved, the ligand binding specificities of the three MSCRAMMSs vary (Figure 3D) [18], [21], [28]. All the bound peptides form into a β-strand paired with the G-strand and pass through the tunnel formed by the N2, N3 and the end of the G-strand (Figure S3). In the ClfB-Fg α_(316–328)_/CK10_(499–512)_ structures, one peptide is bound to one ClfB, in the same orientation as the Fg γ-chain peptide in ClfA and a reverse orientation compared to the Fg β-chain peptide in SdrG (Figure 3D) [21], [28].

In both ClfA-Fg γ and SdrG-Fg β structures, the C-terminus of the N3 domain forms a β-stand G′ (Figure 3D). ClfA^Tyr338^ that is conserved in the structures of SdrE and SdrD (data not shown), forms a hydrogen bond with the amino acid at the end of the G strand (Asn530 in ClfA), thus stabilizing the conformation of the G′ strand (Figure S4). In ClfB, the amino acid at the corresponding position is substituted with phenylalanine (ClfB^Phe328^) (Figure 3A). Comparison of the apo- and ligand-bound form structures of ClfB indicates that the interactions between the ligands and the G strand of N3 play a vital role in the redirection of the C-terminus of N3. ClfB^Arg529^, the last residue in the C-terminus of the G strand in ClfB, interacts with the ligand peptides in both complex structures. ClfB^Asn238^ and ClfB^Arg529^ form a stable hydrogen bond to lock the peptides into the GG′ covered tunnel. Interestingly, although in the ClfB-CK10 structure the G′ strand appears disordered, the ClfB^Asn238-Arg522^ hydrogen bond also exists (Figure 2B), consistent with the DLL model. Taken together, our structures strongly support the DLL model for ClfB-ligand binding.

### Peptides recognition of ClfB

In the crystal structures of the ClfB-Fg α_(316–328)_/CK10_(499–512)_ complexes, both peptides lie down into a tunnel between N2 and N3. The peptides are covered by the C-terminal end of β-strand G (Figure S2). The C-termini of the two peptides have nearly identical conformations, with a turn formed at Fg α^Gly326^ and CK10^Gly510^ (Figure S5). In contrast, the N-termini of the peptides are notably different. A sharp twist at Fg α^Gly318^ allows the N-terminal portion of the peptide to exit the tunnel and point upward. Unlike Fg α_(316–328)_, CK10_(499–512)_ adopts a more extended conformation.

**Figure 4:**
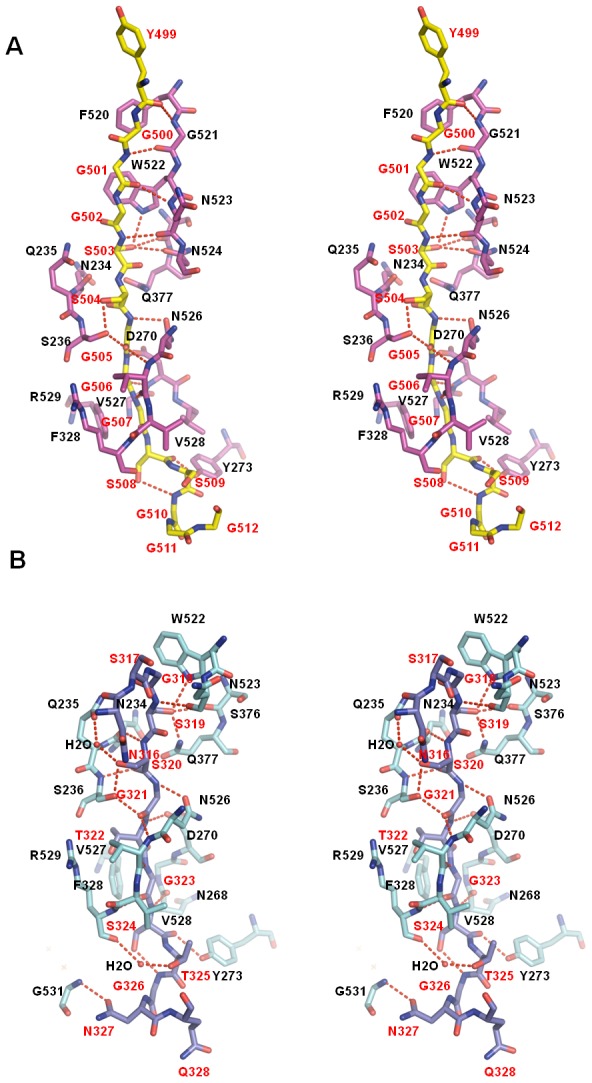
Detailed interactions between the ligand binding of ClfB in the ClfB-CK10/Fg α complexes. A. Detailed interactions between the ligand and ClfB in the ClfB-CK10 complex. The ClfB and CK10 peptides are shown as sticks, colored in magenta and yellow, respectively. The hydrogen bonds are indicated by red dashed lines. The amino acids of ClfB and CK10 are marked with black and red characters, respectively. B. Detailed interactions between the ligand and ClfB in the ClfB-Fg α complex. The ClfB and Fg α peptides are shown as sticks, colored in cyan and slate, respectively. The hydrogen bonds are indicated by red dashed lines. The amino acids of ClfB and Fg α are marked with black and red characters, respectively.

Numerous contacts with distances of less than 4 Å between the protein and the peptides are observed (Figure 4 and Figure S6). The interactions between ClfB with the peptides are primarily mediated through a number of hydrogen bonds. The conserved hydrogen bonds are observed between ClfB and the middle region of the two peptides (Figure 4). Hydrophobic contacts of the middle region of both peptides with the G strand of ClfB_(208–542)_, the loop between β-sheet A, B and the loop between β-sheet C, D of N2 domain also contribute to peptide-protein interactions.

In the ClfB_(208–531)_-CK10_(499–512)_ complex structure, four pairs of hydrogen bonds were observed between the main chains of the peptide and the G strand of N3 domain, resulting in the formation of a parallel β-sheet. Polar groups in side chains of ClfB^Trp522, and Asn524^ in N3 domain form two hydrogen bonds with the hydroxyl group of CK10^Ser503^. The hydroxyl group of CK10^Ser503^ forms the third hydrogen bond with ClfB^Ser376^ of N3 and side-chain hydroxyl group of CK10^Ser504^ forms another hydrogen bond with ClfB^Ser236^ of N2. Residues from the middle region of CK10 interact with ClfB^Ser236, Asp270, and Asn526^ via main chain-main chain hydrogen bonds (Figures 4A and Figure S6A). Hydrogen bonds were also observed between the amino groups of CK10^Ser504, and Gly506^ and side chain hydroxyl or carbonyl groups of ClfB^Asn234, and Asp270^ in the loop region of N2. The carbonyl groups of the C-terminal residues CK10^Ser508, and Ser509^ interact with the side chain hydroxyl group of ClfB^Tyr273^ in the CD-loop of N2 (Figure 4A and Figures S6A, B). The aromatic ring of ClfB^Trp522^ of the G strand of N3 plays an important role in anchoring the N-terminus of CK10 peptide through hydrophobic interactions with CK10^Gly501^ and CK10^Gly502^. The C-terminal segment of the peptide lies in the hydrophobic trench formed by residues of the loop region of N2 and is covered by the G strand of N3 (Figure 4A and Figures S6A,B).

Foster's study demonstrated that substitution of CK10^Gly507^ with the bulky residue tyrosine resulted in loss of interaction of CK10 with ClfB [33]. Structural analysis showed that the space surrounding CK10^Gly507^ is significantly circumscribed by its neighboring residues ClfB^Val528, Gly269, Val271, and Phe328^. Modeling studies (data not shown) indicated that any residue with a side chain would generate steric hindrance and cannot be accommodated in the pocket defined by the above four ClfB residues (Figure 4B and Figures S6C,D).

### Mechanisms of specifically recognizing repeat 5 of Fg α (Fg α5) by ClfB

**Figure 5:**
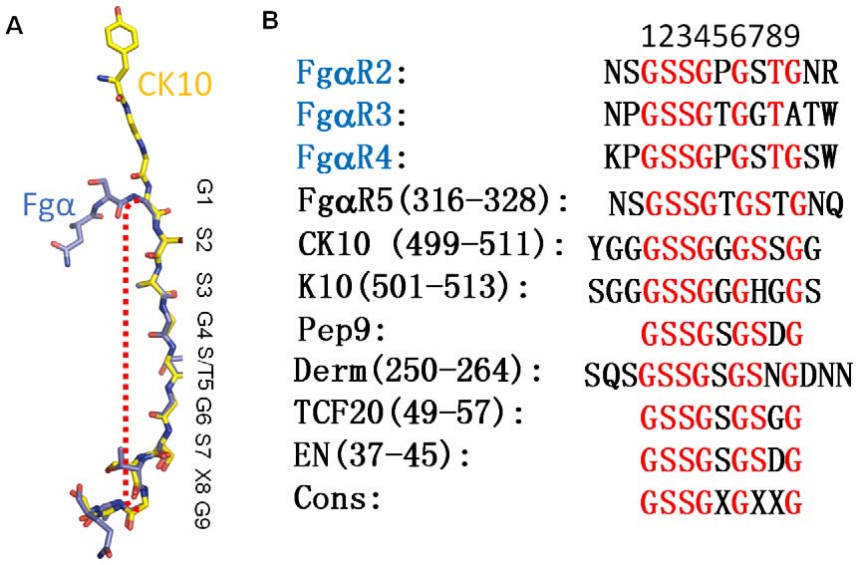
Mechanisms of specifically recognizing repeat 5 of Fg α. A. Superimposition of the Fg α and CK10 peptides. The Fg α and CK10 peptides are shown as sticks, colored in yellow and slate, respectively. Residues highlighted within the boundaries of the red dashed line constitute the segment important for ClfB binding. The consensus amino acids are shown above the peptides. B. Sequence alignment of the repeat 2, 3, 4 and 5 of the Fg α, CK10 (type I cytokeratin 10, residues 473–485 and residues 499–511), K10 (Keratin 10, type I cytoskeletal 10 isoform-1 from *Pan troglodytes*, residues 501–513), Derm (Dermokine, residues 250–264), TCF20 (TCF20, residues 49–57), EN (Engrailed protein, residues 37–45) and the derived peptide 9. The conserved amino acids are shown in red and the consensus sequence is designated below the sequences. The repeat 2, 3 and 4 of the Fg α which have been proved cannot bind to ClfB are indicated in skyblue.

The Fg α C-terminal domain (amino acids 221–610) of human Fg contains ten 13-residue tandem repeats, within which up to eight residues are glycines or serines [34]. Despite the similar sequences among the repeats, only Fg α5 was shown to be recognized by ClfB [18]. The reason for this was proposed to be the presence of proline or arginine residues in the center of the putative Ω loops in the other repeats though the precise underlying mechanism remains unknown [27]. The crystal structures presented here offer an explanation for this observation. Structural comparison of the two complexes revealed that interactions of the peptides with ClfB are primarily mediated through a conserved motif in the peptides: G-S-S-G-S/T-G-S-X-G (Figure 5A). Sequence alignment of the repeats indicates that Fg α5 differs from the other repeats at the 5^th^, 7^th^ and 9^th^ positions (Figure 5B). The hydroxyl group of S/T at the 5^th^ position is involved in hydrogen bonding interactions. On the other hand, the size of the residue at this position is limited by its neighboring residues. Thus, other residues except S/T at this position are expected to compromise the interactions between the repeat and ClfB either because of loss of hydrogen bonding interaction or generation of steric hindrance. The 7^th^ position appears to play a role in maintaining the local conformation of the peptide by forming a γ-turn with the 9^th^ position. In the structure of CK10-ClfB complex solved by V.Ganesh et al., the 7^th^ position was replaced with a histidine residue, suggesting that the residue at this position can be varied (Figure S6). The G_9_ residue was headed to the end of the β-sheet D and the ClfB^Met280^ and ClfB^Pro281^ in N2 limit residues with any side chain which would generate clash against them. In addition, a turn at the G_9_ is required to permit the peptide out of the tunnel, explaining why the repeat 2 with an alanine at this position cannot bind to ClfB (Figure 5B) [18].

### Importance of the GSR motif in recognition by ClfB

After carefully analyzing the sequences and the peptide binding specificities of ClfB, we propose that a small motif G_1_-S_2_-S_3_-G_4_-G/S/T_5_-G_6_-X_7_-X_8_-G_9_ is responsible for ligand binding to the ClfB adhesive domains. Taking the Fg α_(316–328)_-ClfB complex as an example, within this motif, the G_1_ is limited by the side chain of ClfB^W522^ with the limitation of the space and is also required for the Fg α peptide making a turn thus exiting the tunnel. The S_2_ is the most critical residue because it not only forms two hydrogen bonds with the side chains of ClfB^W522^ and ClfB^Q377^ but also binds to the main chain of ClfB^S376^. Similar to the S_2_, the S_3_ forms two hydrogen bonds with the side chain of ClfB^Q235^ and ClfB^S236^ in the N2 domain, which could be replaced by a smaller residue such as alanine. The following residues, especially the G_4_, G/S/T/_5_ and G_6_, are necessary for the formation of the stable protein-peptide complex because they form hydrogen bonds with ClfB and the size of the β-sheet G covering tunnel does not accommodate residues with larger side chains. The S_7_ might play a role in maintaining the local conformation of the peptide by forming a γ-turn with the G_9_. The space for the S_8_ appears to be enough for residues with larger side chains (Figure 5B and Figure S6). Finally, the G_9_ needs to form a turn to allow the peptide out of the tunnel. Thus, the somewhat soft binding trench of ClfB would be able to bind to a series of peptides with this feature.

**Figure 6:**
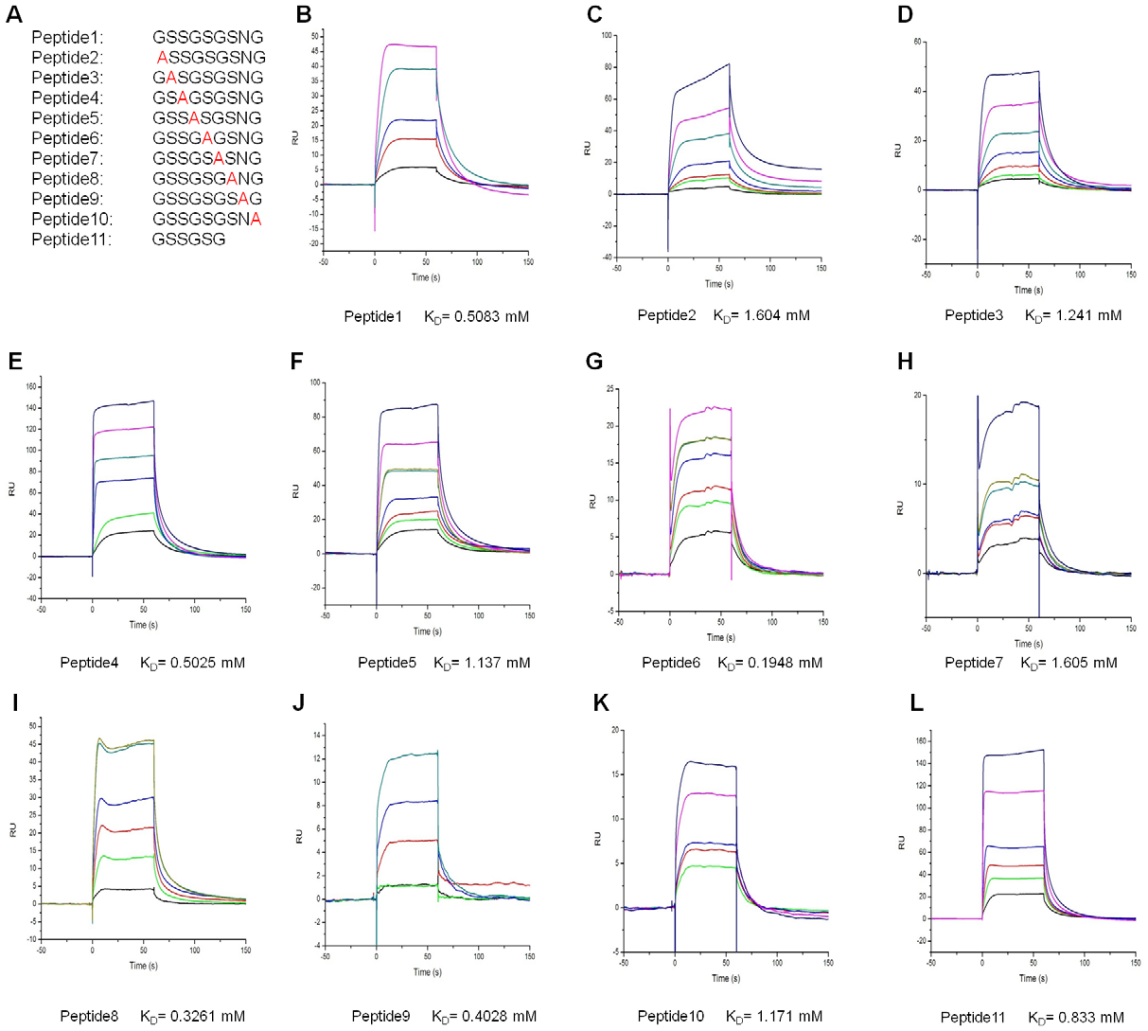
The SPR analysis of the interactions between ClfB and dermokine peptides. A. Panel of dermokine peptides. Peptide 1 corresponds to the 9-residue peptide derived from dermokine protein (253–261). The substituted peptides (Peptides 2–10) have individual amino acids replaced with Ala and peptide 11 is the six amino acid peptide. (B–L). The SPR analysis of the binding between ClfB and Peptides 1–11. Navy, 2000 µM; Magenta, 1000 µM; Dark cyan or dark yellow, 500 µM; Blue, 250 µM; Red, 125 µM; Green, 62.5 µM; Black, 31.25 µM. K_D_ values of individual binding assays are indicated below the sensorgrams.

To further confirm our hypothesis regarding the importance of the nine-amino-acid GSR motif, we did the alanine scan using the SPR (Surface Plasmon Resonance) system with a synthetic 9-residue peptide derived from the GSR motif (GSSGSGSNG). The results are highly consistent with our structural observation and clearly show that the nine-amino-acid peptide is necessary and sufficient for binding to ClfB *in vitro* (Figure 6).

### Dermokine is a potential ligand of ClfB

Our results suggest that proteins carrying the GSR motif are able to bind to ClfB. To find other potential ligands of ClfB, we searched the NCBI protein database for additional proteins containing the sequence of G_1_-S_2_-S_3_-G_4_-G/S/T_5_-G_6_-X_7_-X_8_-G_9_. Three proteins, TCF20, Engrailed protein and Dermokine (Derm) were found to be the hits, out of which Dermokine was evaluated more in detail in this study (Figures. 5 and 6). Dermokine is expressed in many epithelial tissues, localized to intracellular or pericellular spaces and overexpressed in inflammatory diseases. The two major isoforms α and β are transcribed from different promoters at the same locus. Recently, additional transcript variants γ, δ and ε have been identified [35], [36].

**Figure 7:**
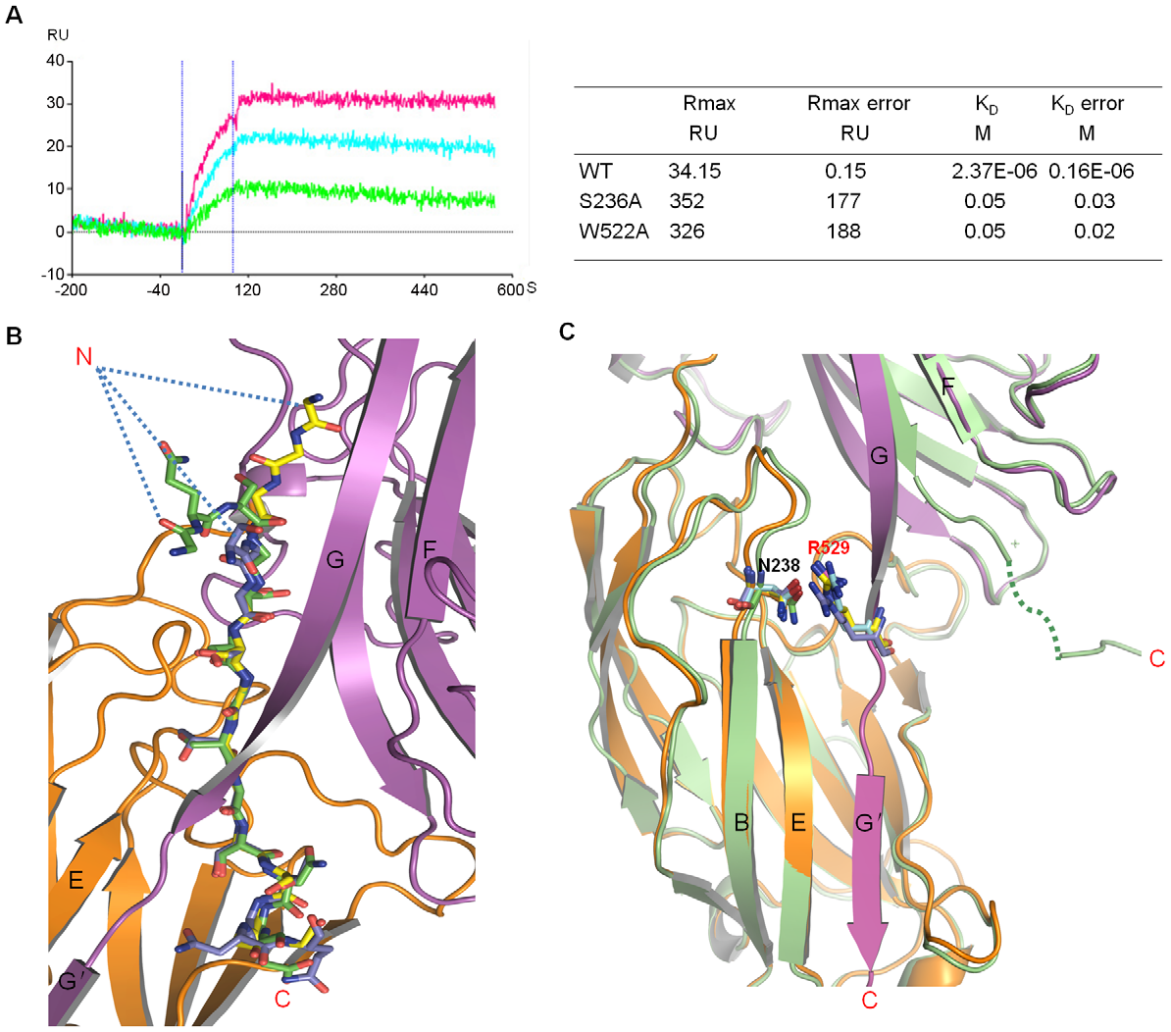
Dermokine is a potential ligand of ClfB. A. Left: Surface plasmon resonance shows the binding of different concentrations of ClfB_(197–542)_ to synthetic peptide 15 from Dermokine immobilized on a Proteon NLC Sensor Chip. Red, 700 µM; blue, 350 µM; green, 87.5 µM. K_D_ was found to be 2.37 µM. Right: kinetic and affinity binding values of the ClfB_(197–542)_ wildtype, S236A or W522A single mutants with Derm15 peptide. B. Comparative close-up view of CK10, Fg α and Derm15 binding to ClfB. The peptides CK10, Fg α and Derm15 are shown as sticks and colored in yellow, slate and green, respectively. The N and C termini are marked. The color schemes of the N2 and N3 domain of ClfB are the same as in Figure 1B. C. Closer view of the superimposition of apo-ClfB and ligand-ClfB complexes. N238 and R529 are highlighted and shown as sticks. The apo-ClfB is colored in lime and the others are in the same color scheme as in Figure 1B.

Firstly, Derm was tested for its interaction with ClfB. To this end, we synthesized a 15-amino-acid-peptide (250–264; GQSGSSGSGSNGDNN, designated as Derm15 hereafter) derived from Derm and then characterized its binding to ClfB using the SPR (Surface Plasmon Resonance) assay. In the assay, ClfB bound to the peptide with a dissociation constant of 2.37 µM (Figure 7A). Interestingly, the results also showed that the Derm peptide interacted with ClfB with slow kinetics, further supporting the DLL model (Figure 7A). To understand the molecular mechanism underlying this interaction, we solved the crystal structure of ClfB_(208–542)_ bound to the peptide at 2.5 Å resolution. As expected, Derm15 interacts with ClfB in a nearly identical manner with Fg α_(316–328)_ and CK10_(499–512)_ (Figure 7 B). The ClfB^Arg529^ forms a hydrogen bond with ClfB^Asn328^ and the C-terminus of N3 forms an extra strand, which is similar as that in the ClfB-Fg α_(316–328)_ and ClfB-CK10_(499–512)_ complexes (Figure 7 C). Mutagenesis studies were conducted to further verify the binding of Derm15 to ClfB. We replaced the residues ClfB^S236, W522^ that participate in interactions with the peptide with alanine respectively. The mutant proteins were purified to homogeneity and tested for their interaction with the Derm peptide using SPR. While the wild type ClfB bound tightly to Derm15, the mutant proteins ClfB_(197–542)_ S236A or W522A exhibited much lower binding affinities with the peptide in mM range (Figure S8). Interestingly, besides the low binding affinities, both mutant proteins exhibited rapid association and dissociation behaviors in the experiments, as compared to the slow association and scarcely any dissociation behaviors observed for the wild type protein. These results indicated that the residues ClfB^S236, W522^ are not only involved in the binding with ClfB, but also participate in stabilizing or “locking” the peptide in place. Collectively, our results strongly support the interaction between ClfB and Derm *in vitro* and suggest that Derm may involve in the infection process and pathogenesis caused by *S. aureus in vivo*.

## Discussion

The colonization of the host nares by the Gram-positive bacterium *S. aureus* is mediated by a family of cell surface proteins which promote its adhesion to the extracellular matrix, that is, the MSCRAMMs [13], [18]. ClfB, as a component of this family protein, has been studied for the past decade and was unique in its multi-functional characteristics, as compared to ClfA and SdrG that only bind to fibrinogens [18], [19], [21], [29].

Consistent with the studies of the SdrG-fibrinogen complex [21], data from this study support the DLL binding mechanism of ClfB with the Fg α/CK10-derived peptides, but not the mechanism suggested in the previous study by V. Ganesh et al. [29]. In their work, due to the absence of the “Latch” procedure observed in the crystal structure, the binding mechanism was ascribed to the “DL” model. However, the structures of ClfB-peptide complexes solved in this study, together with the SPR data, indicate that the DLL model should be the mechanism utilized by ClfB to bind to its ligands. Our results also indicate that the DLL model may be the principal mechanism of MSCRAMM-ligand complexes.

In V. Ganesh et al.'s studies of the ClfB complexes, they proposed a common GSSGXG motif constituting the ClfB binding site [29], which is inconsistent with the previous studies on ClfB. For example, within the ten tandem Fg α repeats, repeat 2, 3, 4 and 5 all contain the GSSGXG motif but only the repeat 5 can bind to ClfB (Figure 5B) [18]. Our structural and the alanine screening analyses demonstrate that a 9-residue peptide G_1_-S_2_-S_3_-G_4_-G/S/T_5_-G_6_-X_7_-X_8_-G_9_ is necessary and sufficient for binding to ClfB *in vitro*. It is therefore predicted that a protein incorporating such a motif is able to interact with ClfB. Indeed, our biochemical assays showed that a Dermokine-derived peptide containing the ClfB binding motif interacted with ClfB (Figures 7B, 7C). Further supporting this prediction, our structural studies revealed that the binding mode of the Dermokine-derived peptide to ClfB is nearly identical with that of the Fg α/CK10-derived peptide (Figure 7B). Collectively, these findings raise a provocative possibility that ClfB might act on multiple targets during *S. aureus* infections. Given the fact that ClfB acts as a key determinant of *S. aureus* nasal colonization, this may not be totally surprising.

Interestingly, Dermokine was first identified as a gene expressed in the suprabasal layers of the epidermis, and more recently, other isoforms of this gene besides its α and β isoforms have also been found. This gene is expressed in various cells and epithelial tissues and over-expressed in inflammatory conditions [35], [36], suggesting that Dermokine might play a role in inflammatory processes since the over-expression of the mediators in immune cell activation characterizes many inflammatory diseases. ClfB is involved not only in the *S. aureus*'s colonization of human nares but also in the diseases caused by this bacterium. Additionally, *S. aureus* has also been implicated in several inflammation processes including corneal inflammation. ClfB's binding to Dermokine raises the possibility that ClfB might play a role in the *S. aureus* caused inflammation and the Dermokine gene's over-expression might serve as biological markers whose products could bind to ClfB and participate in this process. Obviously more investigations are needed to verify ClfB-Dermokine interaction during *S. aureus* infections as well as the biological significance of the interaction.

The characterization of ClfB as a multi-ligand binding protein will be meaningful for the identification of putative substrates and for furthering our understanding of the *S. aureus* infection pathway. Our findings also provide important leads towards the development of new therapeutic agents capable of eradicating *S. aureus* carriage in individuals and efficiently interfering with staphylococcal infection. This is particularly important since new antibacterial strategies are in urgent need to combat the drug resistant bacteria that continuing to emerge [37], [38].

## Materials and Methods

### Cloning, expression and purification of the recombinant proteins

The fragment of the ClfB gene (corresponding 197–542 aa) was amplified by PCR from the *S. aureus* Newman genomic DNA. After digestion with *Bam*HI and *Hind*III (NEB), the amplified genes were cloned into the prokaryotic expression vector pQE32 (GE Healthcare Life Sciences) to produce His-tagged fusion protein and were confirmed by DNA sequencing. The expected protein was expressed in *E.coli* strain BL21 with a high yield. Recombinant His-tagged protein was purified by Ni-affinity column chromatography and ion exchange chromatography. For the purification of protein-peptide complexes, the synthesized peptides were added into the concentrated protein samples at a 10∶1 ratio and further subjected to gel filtration chromatography (Superdex-75 column) using buffer (10 mM Tris-HCl pH 8.0, 150 mM NaCl, 2 mM DTT) on the FPLC system (GE Healthcare Life Sciences). The proteins from different stages of purification (i.e. affinity and gel filtration chromatography) were monitored by SDS-PAGE. The selenomethionine (Se-Met)-substituted ClfB derivative was expressed and purified similarly.

### Crystallization and structure determination

The apo-ClfB and its complexes with different peptides were concentrated to 30 mg/ml in 10 mM Tris-HCl pH 8.0, 150 mM NaCl and 2 mM DTT. Crystals were produced by the hanging-drop vapor diffusion method [39] using sparse-matrix screen kits from Hampton Research (Crystal Screen reagent kits I and II), followed by a refinement of the conditions through the variation of precipitants, pH, protein concentrations and additives.

Crystals were grown at 18°C by mixing 1.1 µl of protein with 1.1 µl of reservoir solution and equilibrating against 200 µl of reservoir solution. The apo-ClfB crystals are grown in 0.2 M LiSO_4_, 0.1 M Tris-HCl pH 8.5, 30% polyethylene glycol 4000 and all the complexes with peptides are grown in 0.1 M sodium citrate tribasic dehydrate pH 5.6, 20% 2-propanol and 20% polyethylene glycol 4000. Similar conditions were used for generation of the crystals of Se-Met-substituted ClfB. Native and Se-SAD data were collected at Shanghai Synchrotron Radiation Facility (SSRF) at a wavelength of 0.919 Å and 0.979 Å respectively using a MAR225 (MAR Research, Hamburg) CCD detector at 100 K and processed with HKL2000 [40]. Further processing was carried out using programs from the CCP4 suite (Collaborative Computational Project, 1994).

The selenium sites were located using SHELXs [41] from the Bijvoet differences in the Se-SAD data. Heavy atom positions were refined and phases were calculated with PHASER's SAD experimental phasing module [42]. The real-space constraints were applied to the electron density map in DM [43]. The resulting map was of sufficient quality for model building of the ClfB molecules in COOT [44]. The structures with other peptides were solved with molecular replacement methods in CCP4 and all the structures were refined with the PHENIX [45] packages. Data collection and structure statistics are summarized in table 1.

### Synthetic peptides

The synthesis and purification of the peptides were described previously [18], [27]. For the following peptides, the amino acid residue numbers are given and the sequences are as follows: peptide from repeat 5 of the C terminus of the α-chain of Fg (NSGSSGTGSTGNQ); a peptide in the tail region of CK10 (YGGGSSGGGSSGG); peptide 15 from Dermokine protein (SQSGSSGSGSNGDNN); The peptide 9 of GSR motif (GSSGSGSNG) and its mutated forms by alanine scan; The six-amino-acid peptide (GSSGSG).

### Surface Plasmon Resonance spectroscopy

Binding of ClfB_(197–542)_ to peptide 15 was assessed by SPR using the ProteOn XPR36 equipment (Bio-Rad Laboratories, Inc.). Each SPR experiment used multichannel detection. The system was equilibrated with buffer (10 mM HEPES pH 7.2, 150 mM NaCl). At each channel, peptide was captured to a ProteOn NLC Sensor Chip (BIO-RAD) at 25°C, using a flow rate of 100 µl/min. This resulted in peptide coupled at response levels of 460 RU. For binding measures, ClfB_(197–542)_ was injected simultaneously at different concentrations at a flow rate of 100 µl/min. The experiments were repeated three times.

The binding affinities between ClfB and the ten 9-amino–acid peptides and the 6-amino-acid peptide were determined by surface plasmon resonance (SPR) using BIAcore T200 instrument (GE Healthcare) at 10°C. The ClfB protein was immobilized to about 5300 Response Unit (RU) on a research-grade CM5 sensor chip in 10 mM sodium acetate, pH 5.0 by standard amine coupling method. The flow cell 1 was left blank as a reference. For the collection of data for affinity analyses, the 11 peptides in a buffer of 10 mM HEPES pH 7.4, and 150 mM NaCl, plus 0.005% (v/v) Tween 20, were injected over the flow cells at various concentrations at a 30 µl/min flow rate. The ligands were allowed to associate for 60 s and dissociate for 120 s. Data were analyzed with the BIAcore T200 evaluation software by fitting to a 1∶1 Langmuir binding fitting model.

## Supporting Information

Figure S1 **The two symmetry-related molecules in the unit cell.** A. Ribbon representation of the two symmetry-related molecules in the unit cell. The two molecules are shown in orange and cyan, respectively. B. Electron densities showing the interaction between N terminus of one molecule and the G strand from the other one in the unit cell. S197 and L198 of the N terminus, F and G strand from the other one are marked.
(TIF)

Figure S2 **The electron density of Fg α and CK10 peptides.** A. Ribbon representation of ClfB_(208–542)_-Fg α_(316–328)_ complex. The peptide is shown in sticks and the 2Fo-Fc map around the peptide contoured at 1.5σ is also shown. The color scheme is the same as in Figure 1B. The N and C-termini of both the protein and the peptide are designated, respectively. B. Ribbon representation of ClfB_(208–531)_-CK10_(499–512)_ complex. The peptide is shown in sticks and the 2Fo-Fc map around the peptide contoured at 1.5σ is also shown. The color scheme is the same as in Figure 1B. The N and C termini of both the protein and the peptide are designated, respectively.
(TIF)

Figure S3 **Surface representation of ClfB_(208–540)_, ClfA_(229–545)_ and SdrG_(276–597)_ showing the peptide “locked” into the molecule.** The surface is color-coded according to negative and positive charge residues that are represented as red and blue. The peptides are shown as sticks. (A), ClfB-Fg α_(316–328)_. (B), ClfB-CK10_(499–512)_. (C), ClfA-Fg γ_(395–411)_. (D), SdrG- Fg β_(6–20)_.
(TIF)

Figure S4 **Cartoon view showing the interactions between the Fg γ peptide with ClfA.** The carbon, oxygen and nitrogen atoms are shown in cyan, red and blue, respectively. The residues of peptide are shown as sticks in magenta. The residues of ClfA are marked in black and those from Fg γ are shown in magenta. The hydrogen bonds are indicated in red dashed lines.
(TIF)

Figure S5 **Closer view of the ligand binding tunnel of ClfB.** A, The N termini of the peptides. ClfB is represented as an electrostatic surface model with negative and positive charges indicated by red and blue, respectively. The Fg α peptide was superposed onto the CK10 peptide and they are shown as sticks in blue and yellow, respectively. B, The C-termini of the peptides. The color scheme is the same as in Figure S5A.
(TIF)

Figure S6 **Detail interaction of the ligands binding.** A. Closer view of the ligand binding tunnel of ClfB in the ClfB-CK10 complex. ClfB is represented as an electrostatic surface model with negative and positive charges indicated by red and blue, respectively. The CK10 peptide is shown as sticks in yellow. The hydrogen bonds are indicated by red dashed lines. B. Schematic representation of the hydrogen bond interactions between ClfB and the CK10 peptide. Hydrogen bonds are shown as dashed lines. The interactions with the CK10 come from strand G in N3 domain, AB- and CD-loops from N2 domain in ClfB. C. Closer view of the ligand binding tunnel of ClfB in the ClfB-Fg α complex. The color scheme is the same as in Figure S6A. The hydrogen bonds are indicated by red dashed lines. D. Schematic representation of the hydrogen bond interactions between ClfB and the Fg α peptide. Hydrogen bonds are shown as dashed lines. The interactions with the Fg α come from strand G in N3 domain, AB- and CD-loops from N2 domain in ClfB.
(TIF)

Figure S7 **The electron density of Derm15 peptide.** Ribbon representation of structures of ClfB_(208–542)_ binding to Derm15 peptide from Dermokine. The peptide is shown in sticks and the 2Fo-Fc map around the peptide contoured at 1.5σ is also shown. The color schemes of both the protein and the peptide are the same as in Figures S2 A and B.
(TIF)

Figure S8 **The ClfB^S236A^ and ClfB^W522A^ single mutants cannot bind Derm15 peptide.** A and B. surface plasmon resonance (SPR) shows the binding of different concentrations of synthetic Derm15 peptide to ClfB_(197–542)_ S236A or W522A single mutants immobilized on a GLH Sensor Chip. Red, 4 mM; green, 2 mM; blue, 1 mM; pink, 0.5 mM; orange, 0.25 mM. C. Kinetic and affinity binding values of the ClfB_(197–542)_ mutants S236A or W522A with Derm15 peptide.
(TIF)
