# Supplementary figures and images for: Crystal Structures Reveal the Multi-Ligand Binding Mechanism of Staphylococcus aureus ClfB
Source: PLoS Pathog. 2012 Jun 14;8(6):e1002751. doi: 10.1371/journal.ppat.1002751 (PMC3375286; doi:10.1371/journal.ppat.1002751)

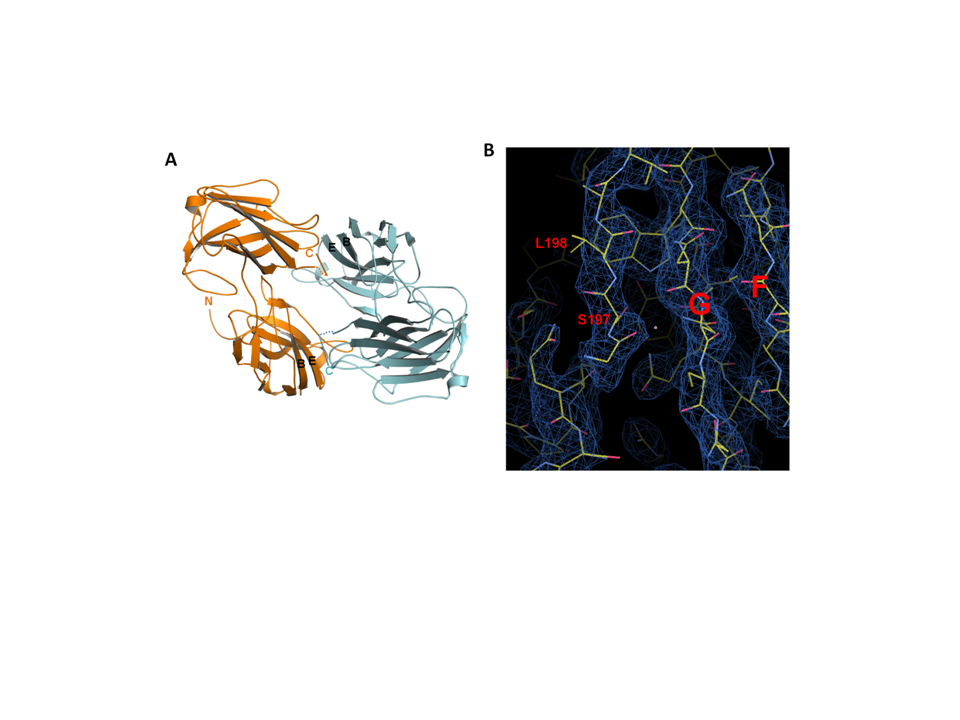

Supplement: Figure S1 — The two symmetry-related molecules in the unit cell. A. Ribbon representation of the two symmetry-related molecules in the unit cell. The two molecules are shown in orange and cyan, respectively. B. Electron densities showing the interaction between N terminus of one molecule and the G strand from the other one in the unit cell. S197 and L198 of the N terminus, F and G strand from the other one are marked. (TIF) [file ppat.1002751.s001.tif]

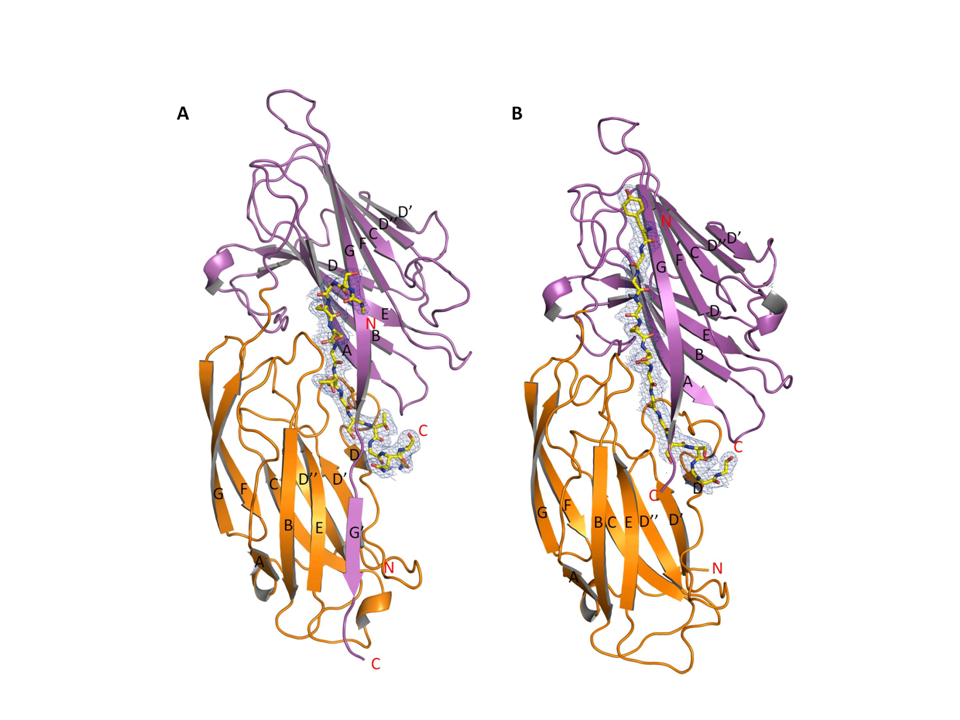

Supplement: Figure S2 — The electron density of Fg α and CK10 peptides. A. Ribbon representation of ClfB(208–542)-Fg α(316–328) complex. The peptide is shown in sticks and the 2Fo-Fc map around the peptide contoured at 1.5σ is also shown. The color scheme is the same as in Figure 1B. The N and C-termini of both the protein and the peptide are designated, respectively. B. Ribbon representation of ClfB(208–531)-CK10(499–512) complex. The peptide is shown in sticks and the 2Fo-Fc map around the peptide contoured at 1.5σ is also shown. The color scheme is the same as in Figure 1B. The N and C termini of both the protein and the peptide are designated, respectively. (TIF) [file ppat.1002751.s002.tif]

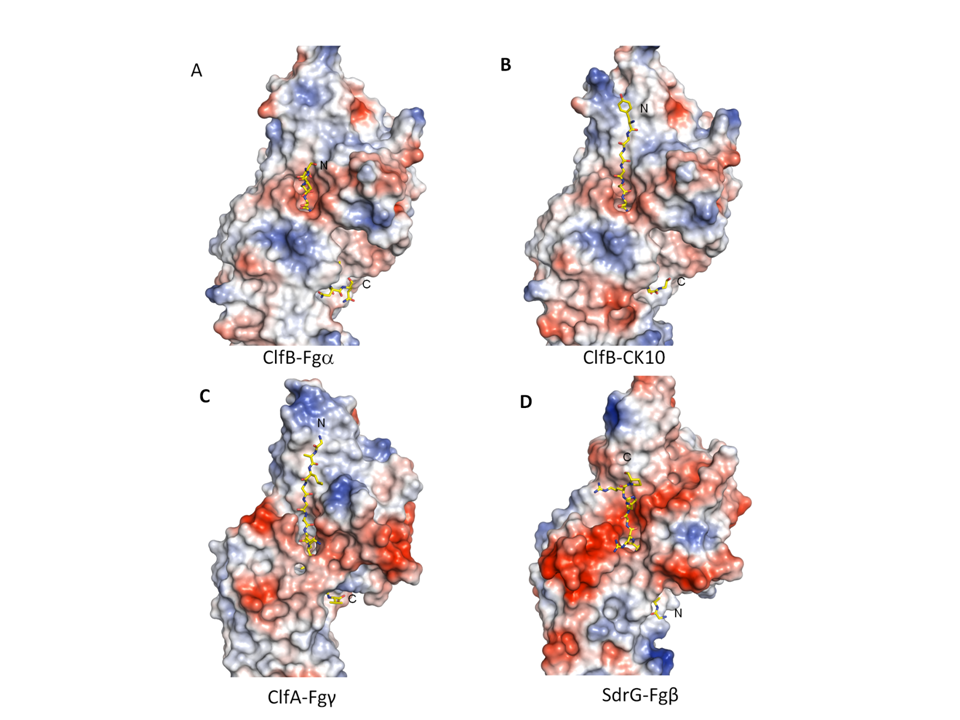

Supplement: Figure S3 — Surface representation of ClfB(208–540), ClfA(229–545) and SdrG(276–597) showing the peptide “locked” into the molecule. The surface is color-coded according to negative and positive charge residues that are represented as red and blue. The peptides are shown as sticks. (A), ClfB-Fg α(316–328). (B), ClfB-CK10(499–512). (C), ClfA-Fg γ(395–411). (D), SdrG- Fg β(6–20). (TIF) [file ppat.1002751.s003.tif]

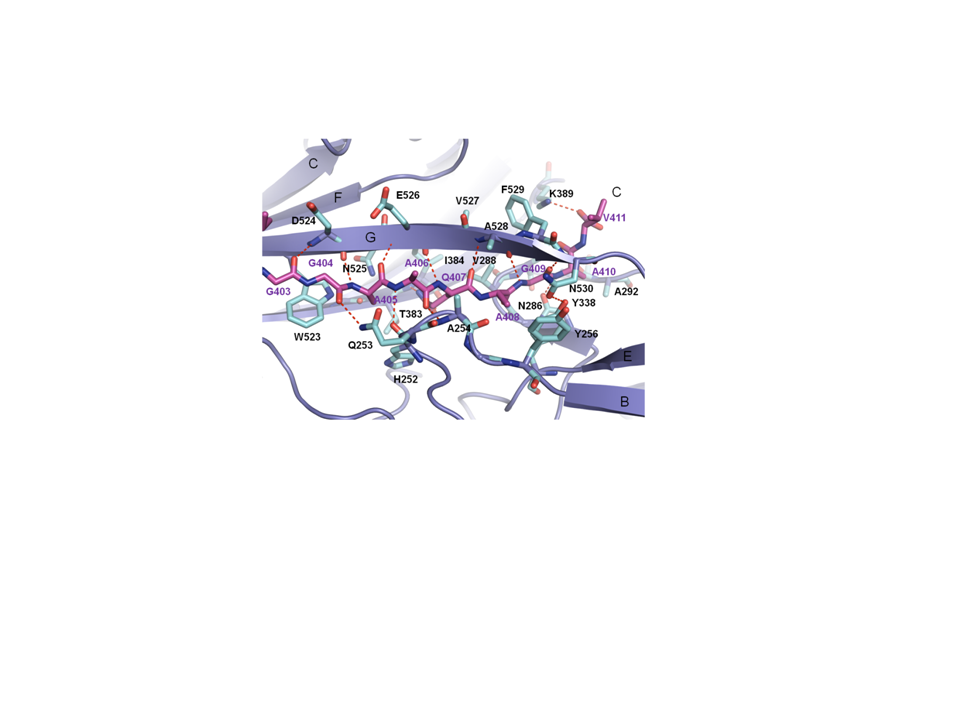

Supplement: Figure S4 — Cartoon view showing the interactions between the Fg γ peptide with ClfA. The carbon, oxygen and nitrogen atoms are shown in cyan, red and blue, respectively. The residues of peptide are shown as sticks in magenta. The residues of ClfA are marked in black and those from Fg γ are shown in magenta. The hydrogen bonds are indicated in red dashed lines. (TIF) [file ppat.1002751.s004.tif]

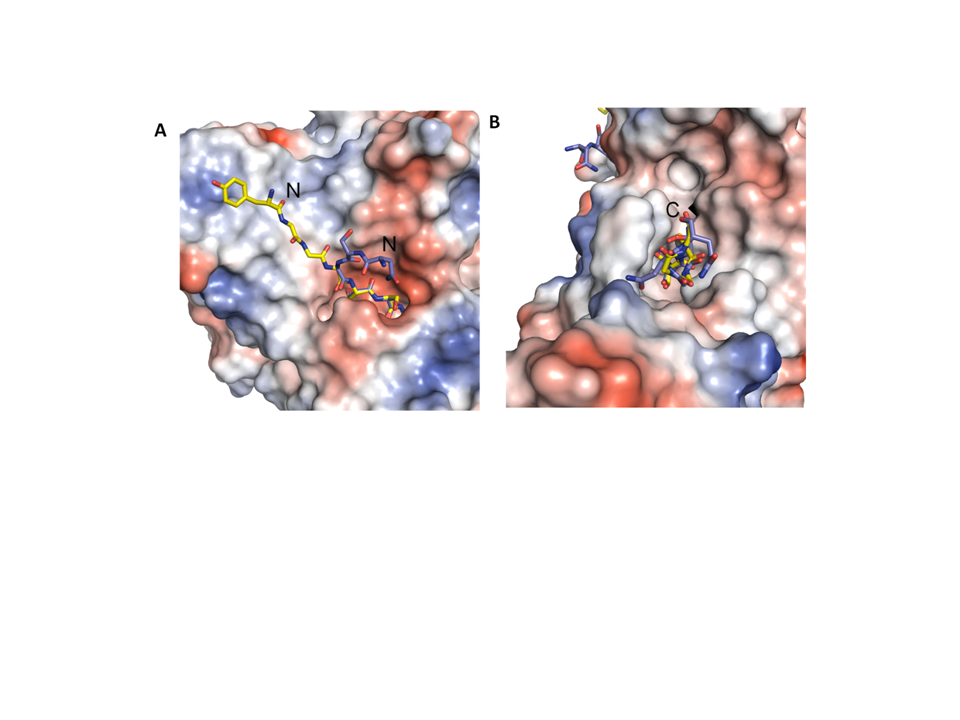

Supplement: Figure S5 — Closer view of the ligand binding tunnel of ClfB. A, The N termini of the peptides. ClfB is represented as an electrostatic surface model with negative and positive charges indicated by red and blue, respectively. The Fg α peptide was superposed onto the CK10 peptide and they are shown as sticks in blue and yellow, respectively. B, The C-termini of the peptides. The color scheme is the same as in Figure S5A. (TIF) [file ppat.1002751.s005.tif]

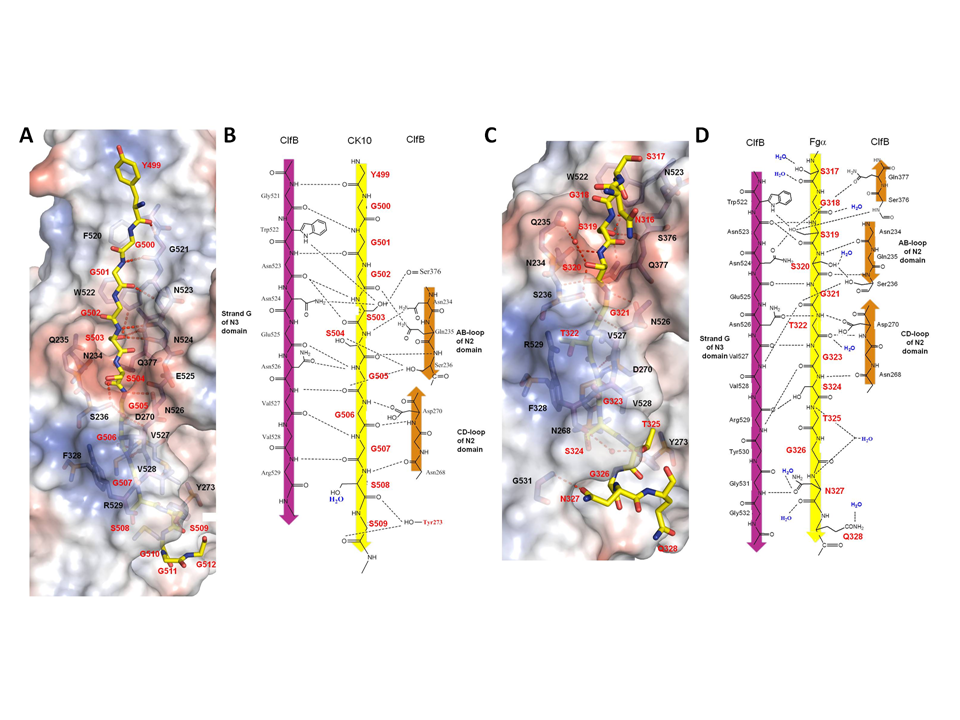

Supplement: Figure S6 — Detail interaction of the ligands binding. A. Closer view of the ligand binding tunnel of ClfB in the ClfB-CK10 complex. ClfB is represented as an electrostatic surface model with negative and positive charges indicated by red and blue, respectively. The CK10 peptide is shown as sticks in yellow. The hydrogen bonds are indicated by red dashed lines. B. Schematic representation of the hydrogen bond interactions between ClfB and the CK10 peptide. Hydrogen bonds are shown as dashed lines. The interactions with the CK10 come from strand G in N3 domain, AB- and CD-loops from N2 domain in ClfB. C. Closer view of the ligand binding tunnel of ClfB in the ClfB-Fg α complex. The color scheme is the same as in Figure S6A. The hydrogen bonds are indicated by red dashed lines. D. Schematic representation of the hydrogen bond interactions between ClfB and the Fg α peptide. Hydrogen bonds are shown as dashed lines. The interactions with the Fg α come from strand G in N3 domain, AB- and CD-loops from N2 domain in ClfB. (TIF) [file ppat.1002751.s006.tif]

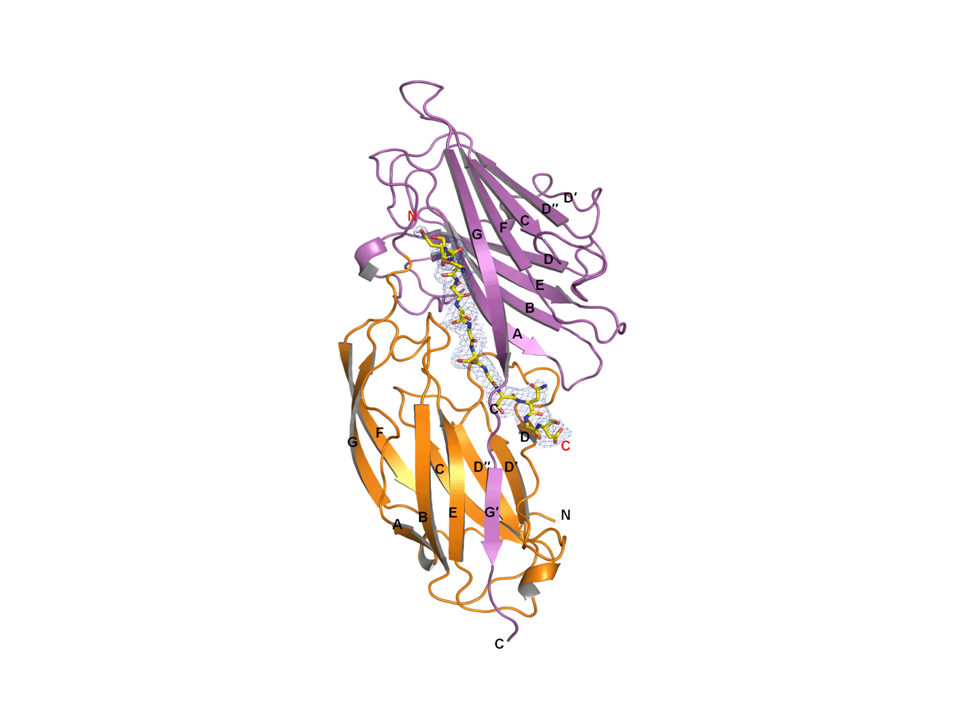

Supplement: Figure S7 — The electron density of Derm15 peptide. Ribbon representation of structures of ClfB(208–542) binding to Derm15 peptide from Dermokine. The peptide is shown in sticks and the 2Fo-Fc map around the peptide contoured at 1.5σ is also shown. The color schemes of both the protein and the peptide are the same as in Figures S2 A and B. (TIF) [file ppat.1002751.s007.tif]

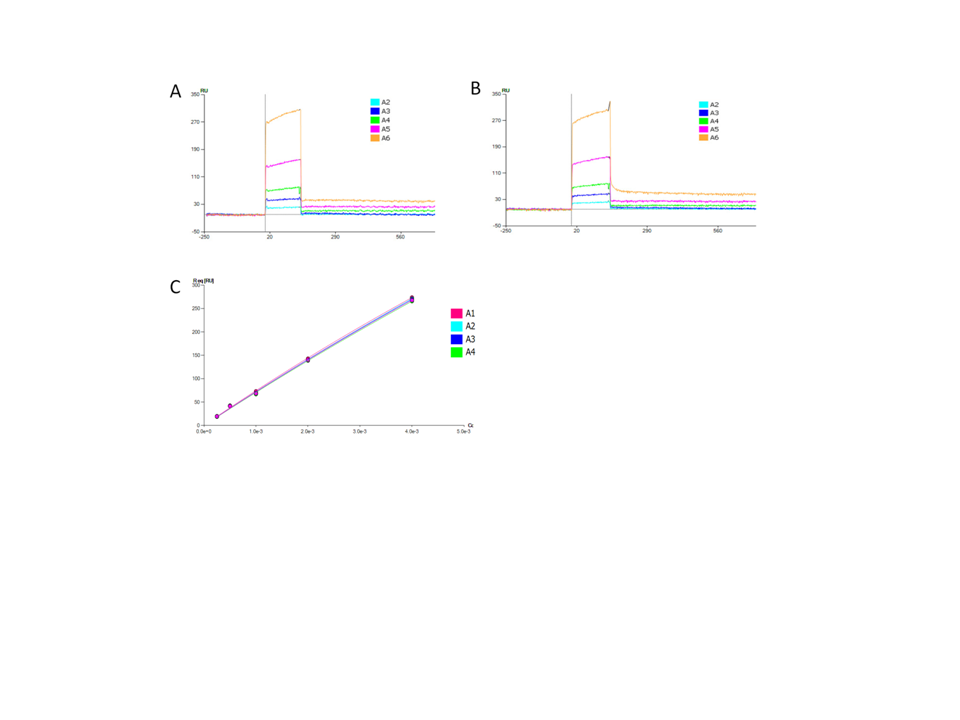

Supplement: Figure S8 — The ClfBS236A and ClfBW522A single mutants cannot bind Derm15 peptide. A and B. surface plasmon resonance (SPR) shows the binding of different concentrations of synthetic Derm15 peptide to ClfB(197–542) S236A or W522A single mutants immobilized on a GLH Sensor Chip. Red, 4 mM; green, 2 mM; blue, 1 mM; pink, 0.5 mM; orange, 0.25 mM. C. Kinetic and affinity binding values of the ClfB(197–542) mutants S236A or W522A with Derm15 peptide. (TIF) [file ppat.1002751.s008.tif]
